# Resolution of a Chikungunya Outbreak in a Prospective Cohort, Cebu, Philippines, 2012–2014

**DOI:** 10.3201/eid2210.160729

**Published:** 2016-10

**Authors:** Anon Srikiatkhachorn, Maria Theresa Alera, Catherine B. Lago, Ilya A. Tac-An, Daisy Villa, Stefan Fernandez, Butsaya Thaisomboonsuk, Chonticha Klungthong, Jens W. Levy, John Mark Velasco, Vito G. Roque, Ananda Nisalak, Louis R. Macareo, In-Kyu Yoon

**Affiliations:** University of Rhode Island, Providence, Rhode Island, USA (A. Srikiatkhachorn);; Armed Forces Research Institute of Medical Sciences Virology Research Unit, Cebu City, Philippines (M.T. Alera, C.B. Lago);; Cebu City Health Department, Cebu City (I.A. Tac-An, D. Villa);; Armed Forces Research Institute of Medical Sciences, Bangkok, Thailand (S. Fernandez, B. Thaisomboonsuk, C. Klungthong, J.W. Levy, J.M. Velasco, A. Nisalak, L.R. Macareo, I.K. Yoon);; Philippines Department of Health National Epidemiology Center, Manila, Philippines (V.K. Roque, Jr.)

**Keywords:** chikungunya, virus, arbovirus, outbreak, active surveillance, prospective cohort, neutralizing antibody, Philippines

**To the Editor:** Chikungunya is a reemerging, mosquitoborne infectious disease caused by chikungunya virus (CHIKV). Its classic manifestations include fever and joint inflammation, which can develop into chronic joint disease. Infections in immunocompromised persons can lead to severe organ involvement and death. Chikungunya outbreaks appear to occur in 2 patterns: 1) spatially and temporally restricted outbreaks in endemic areas (*1*,*2*); and 2) large epidemics that occur periodically every 40–50 years affecting multiple geographic areas (*3*). The mechanisms associated with the initiation of these large outbreaks are not well understood. An A226V amino acid substitution in the virus envelope, which enhances replication in *Aedes albopictus*, a mosquito vector for CHIKV, and expansion of vectors into areas with previously immunologically naive populations are thought to be responsible for some recent epidemics (*4*). In chikungunya-endemic areas, environmental factors such as changes in rainfall and vector densities have been implicated in smaller scale outbreaks. The mechanisms underlying outbreak resolution are not well understood. Herd immunity afforded by exposed persons might play an important role in preventing ongoing virus transmission. Reported seroprevalence rates in affected areas have ranged from 10% to ≈40% after an outbreak (*5*–*7*).

We established an age-structured prospective cohort consisting of persons >6 months of age in Cebu, Philippines, and conducted active surveillance for acute febrile illnesses by making weekly telephone calls or home visits during 2012–2014 (Table). We defined symptomatic chikungunya as an acute febrile illness with CHIKV RNA detected in an acute-phase blood sample or seroconversion detected by CHIKV IgM/IgG ELISA in paired acute/convalescent-phase serum samples. We tested serum samples collected at enrollment, 12 months, and 24 months for neutralizing antibodies by using a CHIKV plaque-reduction neutralization test. Persons identified during active surveillance who had a >4-fold rise in neutralizing antibody titers in the absence of symptomatic chikungunya were considered to have subclinical infection. As reported previously (*8*), the overall incidence of CHIKV infection during the first year of surveillance was 12.32/100 person-years among all cohort members and 16.9/100 person-years among immunologically naive members (defined by the absence of CHIKV neutralizing antibodies at baseline) (Table). Only 19% of infections were symptomatic, and most were accompanied by reported or documented fever without severe joint symptoms. Persons with detectable neutralizing antibodies at the beginning of the surveillance period exhibited no clinical or serologic evidence of CHIKV infection during active surveillance.

**Table:** Incidence of subclinical and symptomatic chikungunya virus infection during 2 years of active surveillance in an age-stratified cohort, Cebu, Philippines, 2012–2014*

Cohort	No. persons/prevalence of neutralizing antibodies at beginning of year, %	No. cases/no. cases per 100 person-years (95% CI)		
		Acute symptomatic infections	Subclinical infections	All infections
Year 1				
All persons with paired serum samples, by age				
6 mo–5 y	203/0.7	5/3.23 (1.23–7.08)	10/6.46 (3.23–11.47)	15/9.69 (5.66–15.59)
6–15 y	201/1.1	8/4.23 (1.99–7.98)	24/12.68 (8.23–18.55)	32/16.91(11/.8–23.56)
16–30 y	200/20.24	2/1.13 (0.23–3.63)	20/11.32 (7.13–17.4)	22/12.45 (8.02–18.51)
31–50 y	204/52.9	4/2.38 (0.79–5.65)	21/12.48 (7.95–18.7)	25/14.85 (9.85–21.57)
>50 y	200/61.06	1/0.56 (0.05–2.6)	12/6.7 (3.66–11.34)	13/7.25 (4.06–12.05)
All ages	1,008/28.0	20/2.3 (1.5–3.49)	87/10.02 (8.08–12.3)	107/12.32 (10.15–14.83)

Only persons with negative neutralizing antibodies at beginning of 1st year, by age				
6 mo–5 y		5/3.26 (1.23–7.4)	10/6.51 (3.34–11.55)	15/9.77 (5.71–15.7)
6–15 y		8/4.27 (2.02–8.6)	24/12.82 (8.43–18.76)	32/17.09 (11.91–23.82)
16–30 y		2/1.42 (0.28–4.56)	20/14.22 (8.96–21.52)	22/15.64 (10.08–23.25)
31–50 y		4/5.02 (1.68–11.94)	21/26.37 (16.81–39.54)	25/31.39 (20.81–45.59)
>50 y		1/1.41(0.13–6.6)	12/16.98 (9.27–28.75)	13/18.39 (10.3–30.55)
All ages		20/3.17 (1.99–4.79)	87/13.77(11.1–16.9)	107/16.94 (13.95–20.38)

Year 2				
All persons with paired serum samples				
6 mo–5 y	148/8.6	2/1.68 (0.33–5.37)	**1/0.84 (0.08–3.91)**	**3/2.51 (0.7–6.71)**
6–15 y	184/18.6	**1/0.63 (0.06–2.93)**	**3/1.88 (0.52–5.03)**	**4/2.51 (0.84–5.97)**
16–30 y	168/35.04	1/0.74 (0.1–5.36)	**4/2.98 (1.0–7.08)**	**5/3.72 (1.41–8.16)**
31–50 y	172/70.4	1/0.62 (0.06–2.87)	**4/2.46 (0.82–5.85)**	**5/3.08 (1.17–6.74)**
>50 y	182/69.7	1/0.61 (0.06–2.86)	**3/1.84 (0.51–4.9)**	**4/2.45 (0.82–5.83)**
All ages	854/42.0	6/0.81 (0.34–1.67)	15/2.03 (1.19–3.27)	**21/2.84 (1.81–4.26)**

Only persons with negative neutralizing antibodies at beginning of 2nd year, by age				
6 mo–5 y		2/1.84 (0.37–5.89)	**1/0.92 (0.08–4.28)**	**3/2.76 (0.76–7.35)**
6–15 y		1/0.77 (0.07–3.6)	**3/2.32 (0.64–6.18)**	**4/3.09 (1.03–7.35)**
16–30 y		1/1.15 (0.1–5.36)	**4/4.6 (1.54–10.94)**	**5/5.75 (2.18–12.6)**
31–50 y		1/2.11 (0.19–9.82)	**4/8.43 (2.82–20.03)**	**5/10.53 (3.99–23.09)**
>50 y		1/2.05 (0.19–9.55)	3/6.15 (1.7–16.4)	4/8.2 (2.74–19.49)
All ages		6/1.42 (0.59–2.93)	**15/3.56 (2.08–5.72)**	**21/4.98 (3.18–7.47)**

Bold indicates significantly different (p<0.05) from incidence observed among the corresponding age groups during the first year of surveillance. Prevalence of neutralizing antibodies at the beginning of the first year was 28% and at the beginning of the second year was 42%.

During the second year of surveillance, 765 cohort members completed all study activities, including undergoing collection of blood samples at the beginning of the study and at the end of the first and second year. The overall incidence of CHIKV infection during the second year (2.84 cases/100 person-years) decreased significantly (p<0.05) compared with the first year (12.32 cases/100 person-years). This change was attributable primarily to a decline in subclinical infections and was observed equally in all age groups. We also observed a decline in incidence of symptomatic infections; however, this difference was not significant, possibly because of the small number of symptomatic cases. The decline in incidence during the second year was also observed when chikungunya–immunologically naive persons were analyzed separately. The prevalence of neutralizing antibodies increased significantly from 28% at the beginning of the first year to 42% at the beginning of the second year. No persons with detectable baseline neutralizing antibodies were infected during the 2-year surveillance period.

Our study documented the resolution of a chikungunya outbreak in a prospective cohort in an endemic setting during 2 years of active surveillance. The duration of this outbreak is consistent with a previous model suggesting that chikungunya outbreaks in the Philippines last ≈1–3 years (*9*). The decline in incidence during the second year coincided with an increase in chikungunya-immune persons at the beginning of the second year, which approached 50%. This seroprevalence rate is higher than the 10%–30% rate reported after major chikungunya outbreaks on the island of Mayotte in the Indian Ocean, on the island of St. Martin in the Caribbean, and in Italy (*5*,*6*,*10*). The higher sensitivity of the neutralization assay in this study compared with the IgM/IgG ELISA used in other studies might have contributed to the higher rate of detection of chikungunya-immune persons. Neutralizing antibodies against chikungunya appear to be long lasting, as indicated by the higher seroprevalence in the older age group in our cohort. A study in Thailand demonstrated the presence of neutralizing antibodies more than a decade after infection (*1*). Although other environmental factors might contribute to outbreak resolution, the absence of infection in cohort members with baseline neutralizing antibodies in our study suggests the protective role of antibodies. A high prevalence of neutralizing antibodies has been documented in a community without any previously reported outbreaks (*1*), suggesting that immunity elicited by subclinical or mildly symptomatic infections might play a role in conferring protection against further transmission, leading to resolution of an outbreak.
